# Single-molecule kinetics and footprinting of DNA bis-intercalation: the paradigmatic case of Thiocoraline

**DOI:** 10.1093/nar/gkv087

**Published:** 2015-02-17

**Authors:** Joan Camunas-Soler, Maria Manosas, Silvia Frutos, Judit Tulla-Puche, Fernando Albericio, Felix Ritort

**Affiliations:** 1 Small Biosystems Lab, Departament de Física Fonamental, Facultat de Física, Universitat de Barcelona, 08028 Barcelona, Spain; 2 CIBER de Bioingeniería, Biomateriales y Nanomedicina, Instituto de Salud Carlos III, 28029 Madrid, Spain; 3 Institute for Research in Biomedicine (IRB Barcelona), Barcelona Science Park, Baldiri Reixac 10-12, 08028 Barcelona, Spain

## Abstract

DNA bis-intercalators are widely used in molecular biology with applications ranging from DNA imaging to anticancer pharmacology. Two fundamental aspects of these ligands are the lifetime of the bis-intercalated complexes and their sequence selectivity. Here, we perform single-molecule optical tweezers experiments with the peptide Thiocoraline showing, for the first time, that bis-intercalation is driven by a very slow off-rate that steeply decreases with applied force. This feature reveals the existence of a long-lived (minutes) mono-intercalated intermediate that contributes to the extremely long lifetime of the complex (hours). We further exploit this particularly slow kinetics to determine the thermodynamics of binding and persistence length of bis-intercalated DNA for a given fraction of bound ligand, a measurement inaccessible in previous studies of faster intercalating agents. We also develop a novel single-molecule footprinting technique based on DNA unzipping and determine the preferred binding sites of Thiocoraline with one base-pair resolution. This fast and radiolabelling-free footprinting technique provides direct access to the binding sites of small ligands to nucleic acids without the need of cleavage agents. Overall, our results provide new insights into the binding pathway of bis-intercalators and the reported selectivity might be of relevance for this and other anticancer drugs interfering with DNA replication and transcription in carcinogenic cell lines.

## INTRODUCTION

Small DNA binders have become powerful tools in biomedicine due to their ability to interact with nucleic acids at specific sites and modulate key processes such as repair, transcription and replication (1–4). Among them, DNA intercalators and bis-intercalators have received continued interest during the last decades due to their particular properties that range from being excellent DNA staining probes (e.g. ethidium, YOYO) to effective anti-proliferative drugs (e.g. doxorubicin, echinomycin) (4,5). From a structural point of view, intercalators are small planar compounds that bind nucleic acids by sliding between two adjacent base pairs and bringing them apart. This process unwinds the DNA double helix and increases its contour length by ∼3.4 Å per intercalator (6). In a DNA bis-intercalator, two of such intercalative moieties are connected by a linker chain. The compound is therefore able to ‘clamp’ DNA by sandwiching two consecutive base pairs between its aromatic rings, distorting and elongating DNA a length twice that of a mono-intercalator.

Although intercalating agents are widely used in anticancer chemotherapy (7), their mechanism of action based on interfering with the transcription and repair machinery makes them also highly toxic causing undesired side effects (8). In order to improve their effectivity, a lot of effort has been put in developing new ligands that (i) exhibit improved sequence selectivity and (ii) form long-lived complexes (9–12). Increased specificity enables recognition and targeting of particular disease-related motifs that can be used for biomedical diagnostics and therapeutics (3,4). Slow off-kinetics of long-lived complexes are related to sustained response of anticancer drugs (10,13). In addition such ligands may also be useful to develop better fluorescent probes with increased imaging times (14). Hence, the development of rapid and accurate methods to elucidate the kinetics and selectivity of small ligands is paramount to advance in the rational design of less toxic and more effective compounds.

Single-molecule force-spectroscopy techniques have proven especially well-suited to investigate the structural and mechanical perturbations that intercalators induce to the DNA double helix (15,16), succeeding in characterizing the binding of very weak ligands (15). This is due to the fact that force spectroscopy uses molecular extension as the natural reaction coordinate and allows to apply force to shift the chemical equilibrium of binding. So far, most experiments have focused on deriving equilibrium information of binding (e.g. affinity) (15–18), whereas only recently attention has been paid to non-equilibrium experiments from which kinetic rates might be inferred (19–21). Moreover, despite the ubiquitous use of DNA intercalators in single-molecule experiments, there is still controversy in some fundamental aspects such as the effects of intercalation on the elasticity of dsDNA (16–17,21–23) and the existence of binding kinetic intermediates (20,23). Similarly, other key parameters such as sequence specificity have been scarcely addressed with these techniques, and still rely on classical footprinting assays that are time-consuming and complex to set up and interpret (24–26).

**Figure 1.**
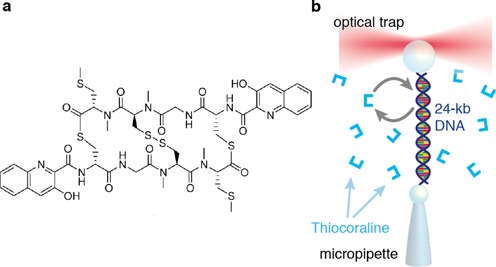
Thiocoraline bis-intercalation using optical tweezers. (**a**) Thiocoraline structure. (**b**) Scheme of the DNA stretching experiments in the optical tweezers setup.

In this work we use optical and magnetic tweezers to study the anticancer bis-intercalator Thiocoraline (Figure 1a) (27), fully characterizing the thermodynamics, kinetics and selectivity of binding using a combination of DNA stretching and unzipping assays (28). A clear understanding of these properties was lacking due to the low solubility of this drug (29,30), making measurements difficult and leading to limited and conflicting results (31,32). We find that Thiocoraline bis-intercalates DNA with an extremely slow dissociation constant that, interestingly, decreases steeply with force. This makes possible to measure the elastic properties of intercalated DNA by performing non-equilibrium pulling experiments in which the binding fraction of ligand remains quenched. We show that bis-intercalation does not modify DNA persistence length whereas the stretch modulus does increase with the bound fraction. A series of kinetic experiments demonstrates the existence of a mono-intercalated intermediate state that contributes to the long lifetime of the complex. Finally, we develop a single-molecule footprinting assay based on DNA unzipping, in which we show that at low concentrations the peptide binds DNA in a sequence-specific manner, with a preference to clamp CpG steps flanked by A-T base pairs. Such information is difficult to obtain in bulk assays and cannot be accessed in usual single-molecule stretching assays.

We propose that this sequence selectivity together with the force-dependent off-rate is an important feature of the mechanism of action of this subnanomolar drug (IC_50_ ∼ 200 pM). On a broader perspective, we provide new insights into the general mechanisms by which bis-intercalators bind DNA and their kinetic stability. The proposed single-molecule approach will be of great utility to quantify essential parameters of the specific and non-specific binding modes of ligands that target DNA (5,25).

## MATERIALS AND METHODS

### Optical tweezers setup

Optical tweezers experiments are performed with a miniaturized dual-beam setup described elsewhere (33). Briefly, a single optical trap is created by focusing two counter-propagating laser beams into a spot (λ = 845 nm, *P* = 200 mW) in a microfluidics chamber. Forces in an optically trapped bead are directly determined from the change in light momentum by measuring the deflection of the exiting laser beams with Position Sensing Detectors (PSDs) (33). The extension and force applied to the molecule can therefore be recorded at 1-kHz rate, and a resolution of 0.1 pN is achieved. Experiments are performed by tethering a single-DNA molecule between two polystyrene beads. One of them is confined in the optical trap, whereas the other is subjected by air suction in the tip of a glass micropipette. Tethers are made by differentially labeling each end of the DNA molecule with several biotins or digoxigenins, which bind to streptavidin- or anti-digoxigenin-coated beads, respectively. In pulling experiments, the relative molecular extension is inferred by subtracting the trap compliance (*F*/*k*) to the relative displacement of the optical trap (*k*_trap_ = 70 pN/μm). The offset to determine the absolute molecular extension is obtained from video microscopy measurements of the bead-to-bead distance. Experimental force–extension curves (FECs) and kinetic traces were recorded at 1 kHz and filtered to 10 Hz.

### Magnetic tweezers assays

Magnetic tweezers experiments are performed with a PicoTwist magnetic tweezers instrument (www.picotwist.com). DNA molecules are tethered between a glass surface treated with anti-digoxigenin antibody (Roche) and a 1-μm streptavidin-coated Dynal magnetic bead (Invitrogen). DNA molecules are manipulated and stretched by capturing the bead in a magnetic trap generated by a pair of permanent magnets. The applied force is controlled by varying the distance from the magnets to the sample. Video microscopy is used to track the position of the magnetic bead in three dimensions with nanometer resolution at 30 Hz, from which the extension of the DNA molecule and the strength of the stretching force are deduced (34).

### Reagents and synthesis of DNA molecular constructs

Thiocoraline was provided by Pharmamar as lyophilized product and dissolved in DMSO as a 1-mM stock solution, which was aliquoted and stored at −20°C. Sample concentration was checked spectrophotometrically and with HPLC. Aliquots were thawed as needed and dissolved in working buffer, adjusting the DMSO concentration to 2%. All experiments were performed in TE buffer (Tris 10 mM, ethylenediaminetetraacetic acid 1 mM) pH7.5, 100-mM NaCl, 0.01% NaN_3_. Experimental controls with drug-free buffer were performed by supplementing the buffer with 2% DMSO.

The synthesis protocols for the 24-kb DNA molecule for stretching experiments, and the 6.8-kb DNA hairpin for unzipping experiments, are described in detail in (28). The 480-bp hairpin for DNA footprinting experiments is synthesized from a plasmid that contains the sequence of interest embedded between the restriction sites of Tsp45I and TspRI. This region of the plasmid is polymerase chain reaction amplified and the product is digested with the two enzymes. A set of oligonucleotides is designed to create the handles structure on the TspRI-digested end following the same approach as explained in (28). Similarly, an oligonucleotide complementary to the Tsp45I end that folds into the end-loop structure is annealed and ligated to create the final hairpin structure. A final gel purification step is added to remove competing structures.

### Analysis of non-equilibrium pulling experiments: elasticity and thermodynamics

Reversible DNA pulling curves at varying Thiocoraline concentrations (0–1000 nM) with optical tweezers (magnetic tweezers) were fitted to the extensible (inextensible) Worm-like chain (WLC) model in the force range 0–40 pN (0–10 pN) using the Marko–Siggia interpolation formula

(1) \begin{eqnarray*} F(l)&=\frac{\text{k}_{\text{B}}T}{L_p}\left( \frac{1}{4(1-l)^2}-\frac{1}{4}+l+\sum _{i=2}^{i\le 7}\alpha _i l^i\right) \nonumber \\ l&=x/l_0-F/S \text{(extensible)} \nonumber \\ l&=x/l_0 \text{(inextensible)} \end{eqnarray*}

where *x* is the molecular extension (end-to-end distance), *l*_0_ is the contour length, *L*_*p*_ is the persistence length, *S* is the stretch modulus and α_*i*_ are the polynomial coefficients determined in (35). Before performing the pulling cycles to measure the metastable FECs, the fraction of bound intercalator was equilibrated to its low force value by setting the force feedback at 2 pN for 5 min. We then performed a set of pulling cycles at a pulling speed (*v* = 1.5–3 μm/s) in which the metastable FECs were fully reversible in the investigated range of forces. For each tethered molecule we determined the *l*_0_, *L*_*p*_ and *S* values by fitting a set of consecutive pulling cycles (typically more than 5). The reported values at each concentration are the average of a set of different molecules (*N* ≥ 7).

**Figure 2.**
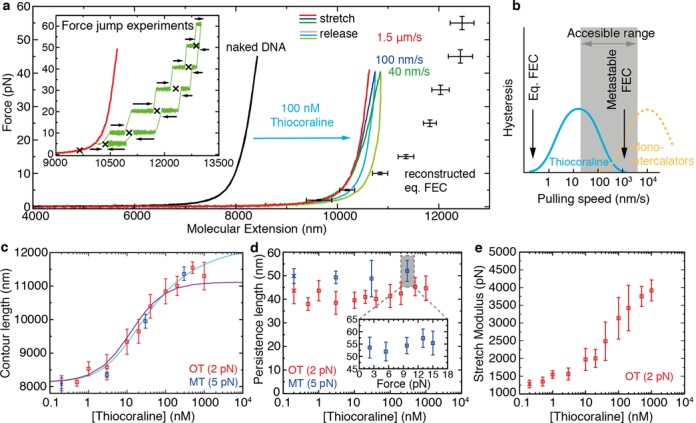
FECs of bis-intercalated DNA and elasticity measurements from metastable FECs. (**a**) DNA pulling curves before (black) and after flowing 100-nM Thiocoraline at varying pulling speeds: 1.5 μm/s (red), 100 nm/s (blue), 40 nm/s (green). Dark (light) colors correspond to the stretching (releasing) parts of the cycle. The equilibrium FEC (black points, mean ± SD, *N* ≥ 5) is recovered by performing force jump experiments. Inset: in force-jump experiments the relaxation of the molecular extension (green) is measured at constant force values starting from different initial conditions in bidirectional experiments (arrows). The equilibrium extension at each force (crosses) can then be inferred. A metastable FEC (1.5 μm/s) is shown in red as a reference. (**b**) Schematics of the hysteresis observed in FECs as a function of pulling speed. For the experimental conditions tested, Thiocoraline (cyan) shows an optimal range to measure metastable FECs, as compared to mono-intercalators (yellow) that reach equilibrium over shorter timescales. (**c**) Contour length (*l*_0_) of DNA as a function of [Thiocoraline] in optical (red squares) and magnetic tweezers (blue squares) experiments. The optical tweezers data are fitted to an MGVH binding isotherm (cyan; Equation. (3)) to obtain the equilibrium affinity and binding-site size at low force (*K*_*d*_ = 77 ± 13 nM, *n* = 3.84 ± 0.12 bp). The theoretical prediction by a three-state model (without free parameters) is shown in purple (see the ‘The kinetic off-rate (*k*_off_) reveals a binding intermediate’ section). (**d**) Persistence length (*L*_*p*_) of DNA as a function of [Thiocoraline]. *L*_*p*_ remains independent of Thiocoraline concentration both in optical tweezers (red) and magnetic tweezers (blue) experiments. Inset: *L*_*p*_ remains also unaffected when the bound fraction is changed by equilibrating the molecule at a different force. (**e**) Stretch modulus (S) of DNA as a function of [Thiocoraline]. *S* monotonically increases with [Thiocoraline]. For panels (c)–(e) values are reported as mean ± SD (*N* ≥ 7 independent molecules), and are determined by fitting metastable FECs (*v* = 1.5–3 μm/s) to the extensible (optical tweezers) or inextensible (magnetic tweezers) WLC model. Reference values from experiments without Thiocoraline for each technique are shown at the lowest concentration (red and blue crosses).

The elongation of the DNA molecule at each Thiocoraline concentration ($l_0^{\text{[Thio]}}-l_0^{\text{DNA}}$) is directly proportional to the number of intercalators bound to the molecule, and to its equilibrium binding fraction (*ν*):

(2) \begin{eqnarray*} \nu =\frac{\# \text{ bound ligands}}{\# \text{ of DNA bp}}=\frac{l_0^{\text{[Thio]}}-l_0^{\text{DNA}}}{\Delta l_{0}N_{\text{bp}}}=\frac{l_0^{\text{[Thio]}}-l_0^{\text{DNA}}}{2l_0^{\text{DNA}}} \end{eqnarray*}

where $l_0^{\text{DNA}}=N_{\text{bp}}d$ (*d* being the base-pair rise, 0.34 nm) and Δ*l*_0_ the DNA elongation induced by one bis-intercalator. We have assumed that each bis-intercalator elongates the DNA molecule a distance twice *d* (Δ*l*_0_ = 2*d*) (6,15). For ligands that bind non-specifically and non-cooperatively to a one-dimensional lattice such as DNA, the McGhee Von–Hippel (MGVH) model (36) predicts that the equilibrium binding fraction satisfies the implicit equation:

(3) \begin{equation*} \nu =\frac{[L]}{K_d}\frac{\left( 1-n\nu \right)^n}{\left(1-n\nu +\nu \right)^{n-1}} \end{equation*}

where *K*_*d*_ is the ligand affinity constant, *n* is the binding-site size (number of bp covered by each ligand) and [*L*] is the ligand concentration. By combining Equations (2) and (3) we could fit the titration results (Figure 2c) and obtain the equilibrium affinity constant *K*_*d*_ and binding-site size *n* of Thiocoraline. A Levenberg–Marquadt algorithm was used both for the WLC and MGVH fitting routines.

### Wash-off experiments and kinetic rates

Wash-off experiments were performed by replacing the buffer in the microfluidics chamber (containing Thiocoraline) with a scavenger DNA solution that created a fast depletion of ligand. The DNA solution was prepared by digestion of λ-DNA with a blunt-ended restriction enzyme (EcoRV) that leaved fragments sized 250 bp–6 kb. In this way, a randomly assorted distribution of sequences equivalent to those in the tethered DNA molecule was obtained. The digested DNA was dissolved to a 10-μM bp concentration in the same buffer as the ligand samples (TE buffer 100-mM NaCl, pH7.5) and flowed into the experimental area using an auxiliary capillary glass tube. A low flow was maintained during the experiments (*F*_drag_ ≤ 2 pN) to ensure ligand removal. The unbinding reactions were fitted to single- and double-exponential functions.

### Statistical analysis of footprinting experiments

First, the experimental force–distance curves (FDCs) were aligned to a theoretical prediction based on the nearest-neighbor model (33). The elastic parameters (*l*_*k*_, *S*) used to model the single-stranded DNA (ssDNA) were obtained from an extensible freely jointed chain (FJC) fit to the relaxation curve of the fully unfolded state (*n* = 480 bp) and were in excellent agreement with previous results for the same ionic condition (37). A small calibration correction (below 5%) on the experimentally measured distance and force was allowed to fully overlap the theoretical and experimental FDCs between the initial rip (*n* = 27 bp) and the fully unzipped state (*n* = 480 bp).

For every data point (*x*_exp_, *f*_exp_) of an FDC, we then determined the most probable apparent number of open base pairs (*n*) by finding the theoretical curve that passed closest to that point at the force *f*_exp_:

(4) \begin{equation*} \left| x_{\rm exp}-x_{\text{th}}\left( n, f_{\rm exp} \right) \right|=\min _{n^*} \left(\left| x_{\rm exp}-x_{\text{th}}\left( n^*, f_{\rm exp} \right) \right|\right). \end{equation*}

The theoretical extension (*x*_th_(*n, f*_exp_)) was determined from the different contributions to the total distance:

(5) \begin{equation*} x_{\text{th}}\left( n, f_{\text{exp}} \right)=x_{\text{h}}\left( f_{\text{exp}} \right)+x_{\text{b}}\left( f_{\text{exp}} \right)+x_{\text{ssDNA}}\left( n, f_{\text{exp}} \right) \end{equation*}

where *x*_b_ is the displacement of the bead from the center of the optical trap, *x*_h_ the extension of the 29-bp dsDNA handles and *x*_ssDNA_ the extension of released ssDNA during unzipping. The term *x*_ssDNA_(*n, f*_exp_) is the only term that depends on the number *n* of unzipped base pairs, and is modeled with an FJC of 2*n* bases with the aforementioned elastic parameters. The other terms do not depend on *n* and are modeled as *x*_b_ = *f*_exp_/*k*_trap_ (where *k*_trap_ is the stiffness of the optical trap) and *x*_h_ using a WLC (Equation (1)) with the generally accepted parameters for double-stranded DNA (dsDNA) (28).

The histogram of *n* for an unzipping curve and the following rezipping curve was then plotted, and binding events identified as extra peaks present in the unzipping histogram. A correct matching between the other regions of the histogram was checked, and finally, the position of the binding peaks was determined from the mean value of a Gaussian fit. The binding positions *n* determined in this way are then correlated to the DNA sequence of the hairpin, and the normalized probability of binding for each sequence *B*_*i*_ is determined as

(6) \begin{equation*} p_{\text{norm}}(B_i)=\frac{N_i/S_i}{\sum _i N_i/S_i} \end{equation*}

where *N*_*i*_ is the number of binding events observed at the sequence *i* and *S*_*i*_ is the number of times that the sequence is found in the DNA hairpin (i.e. degeneracy).

## RESULTS

### FECs of DNA bis-intercalation

We pulled single-dsDNA molecules (half λ-DNA, 24 kb) in the presence of Thiocoraline in the optical tweezers setup (Figure 1b). DNA is tethered between two beads and stretched at a low force (2 pN) until it reaches its equilibrium extension. The molecule is then repeatedly pulled by moving one bead relatively to the other, and the extension measured as a function of the applied force. In this way we recorded FECs in the range 0–40 pN.

At a concentration of 100 nM, the intercalated DNA has a molecular extension significantly longer (∼30%) than that of naked dsDNA (Figure 2a, red and black, respectively), as expected from an intercalative binding mode. Notably, DNA stretching experiments performed at varying pulling speeds show a hysteresis that increases with decreasing pulling speed, whereas hysteresis is not observed at the fastest speed of 1.5 μm/s (Figure 2a, red, blue, green and Supplementary Movie SV1). The observation of hysteresis in DNA pulling curves was previously reported for the bis-intercalator dye YOYO-1 (23), and is caused by the fact that the affinity constant and kinetic rates are force-dependent. It has been shown that the affinity constant of DNA intercalators increases exponentially with applied force (15), and therefore at equilibrium more ligands are bound at higher forces. In a typical pulling cycle the force is increased from a low value (2 pN) up to a high value (40 pN) and then decreased again, enhancing intercalation during a pulling cycle. We identify two relevant timescales in a pulling experiment: the equilibration time of the binding reaction (*τ*_eq_) and the observational time (the duration of a pulling cycle, *τ*_obs_). If pulls were done slowly enough (*τ*_obs_ > *τ*_eq_), the equilibrium FEC would be obtained. In this case we would expect the fraction of bound ligands to increase with force. In the opposite regime (*τ*_obs_ ≤ *τ*_eq_), the molecular extension cannot relax to its equilibrium value at each force, and a positive hysteresis is observed (blue and green FECs in Figure 2a). Remarkably enough, if the pulling speed is increased (*v* > 1 μm/s, red) metastable FECs are measured, where non-equilibrium but reversible curves are obtained. Along these metastable FECs the number of bound intercalators remains quenched, and the FEC does not show hysteresis (Figure 2b). To the best of our knowledge, this metastable state was not attainable with previously studied mono-intercalators and bis-intercalators due to their faster kinetic rates. In the following section, we will exploit metastability to determine the elastic properties of bis-intercalated DNA for a fixed fraction of ligand.

On the other hand, the slow kinetics of Thiocoraline does not allow us to obtain an equilibrium FEC using a standard pulling protocol (Figure 2b). To do so, we performed force-jump experiments in the range 5–60 pN (Figure 2a, inset). In these experiments, the molecule is maintained at a constant force using force-feedback, and the relaxation of the molecular extension is measured at each force in bidirectional experiments (black arrows in Figure 2a, inset). Relaxation curves of force-jump experiments are well described by a single-exponential function (Supplementary Section S1) and their asymptotic value can be used to determine the equilibrium extension at each force (Figure 2a, black points). A comparison between the reconstructed equilibrium FEC and the metastable FEC (Figure 2a, red curve and black points, respectively) illustrates the pronounced effect of force in shifting the binding equilibrium of Thiocoraline. Pulling experiments performed up to a higher stretching force of 70 pN show that Thiocoraline binds DNA at forces above the overstretching transition (Supplementary Section S2).

### Binding thermodynamics and elastic properties of bis-intercalated DNA

The slow kinetics of Thiocoraline makes possible to determine the elastic properties of intercalated DNA in conditions of metastability for a fixed fraction of bound ligand. In our experiments, such fraction is initially equilibrated at a force of 2 pN and the molecule is repeatedly pulled up to 40 pN fast enough to have that fraction quenched throughout the pulling cycle. We performed pulling experiments over 3 decades of ligand concentration (Supplementary Figure S3) and fitted the metastable FECs to the extensible WLC model (see the Materials and Methods section). The contour length increased with Thiocoraline concentration (Figure 2c) reflecting the varying fraction of bound intercalator (6). A fit of the experimental data to the MGVH binding isotherm yielded a binding affinity *K*_*d*_ = 77 ± 13 nM and a binding-site size *n* = 3.84 ± 0.12 bp (see the Materials and Methods section) (15,36). The latter is in good agreement with the values previously determined for other DNA bis-intercalators and from X-ray crystallography (18,38–39).

Fits to the WLC model also yielded the persistence length (*L*_*p*_) and stretch modulus (*S*) of a bis-intercalated DNA molecule. The persistence length remained constant within experimental errors over the whole range of concentrations investigated (Figure 2d). This is in contrast with previous studies for other mono- and bis-intercalators in which a strong decrease of the persistence length with ligand concentration was reported (15–16,22–23,40). In these works, equilibrium FECs were used to obtain the elastic properties of intercalated DNA. However, in the equilibrium ensemble, the fraction of intercalated drug changes as force is increased, leading to a continuously changing contour length within a pulling cycle, thereby giving lower effective persistence lengths in the fits. A constant persistence length has also been reported for YOYO-1 in recent studies performed at low forces (*F* < 10 pN) and low salt condition (10 mM) where force-dependent intercalation is negligible (17). We confirmed our observation by performing experiments with a magnetic tweezers setup in the low-force regime (0.01–10 pN), obtaining equivalent results (Figure 2d, blue). Furthermore, we performed experiments in which the fraction of bound intercalator was increased by equilibrating the molecule at a higher initial force (Figure 2d, inset), finding again that persistence length is independent of the fraction of intercalated drug regardless of the method used to increase the binding ratio (i.e. ligand concentration or force). Finally, from our high-force (*F* > 10 pN) measurements we also obtained the stretch modulus of bis-intercalated DNA, finding a systematic increase in the stretching rigidity with ligand concentration (Figure 2e and Supplementary Figure S4). A fit of the reconstructed equilibrium FEC shown in Figure 2a to the WLC model gives instead *l*_0_ = 12200 ± 200 nm, *l*_*p*_ = 8.1 ± 0.6 nm, *S* = 745 ± 320 pN, where it can be observed that the apparent persistence length and stretch modulus are markedly reduced (and the contour length increased) due to the effective effect of force-induced intercalation.

### Total kinetic rate and direct off-rate measurements

**Figure 3.**
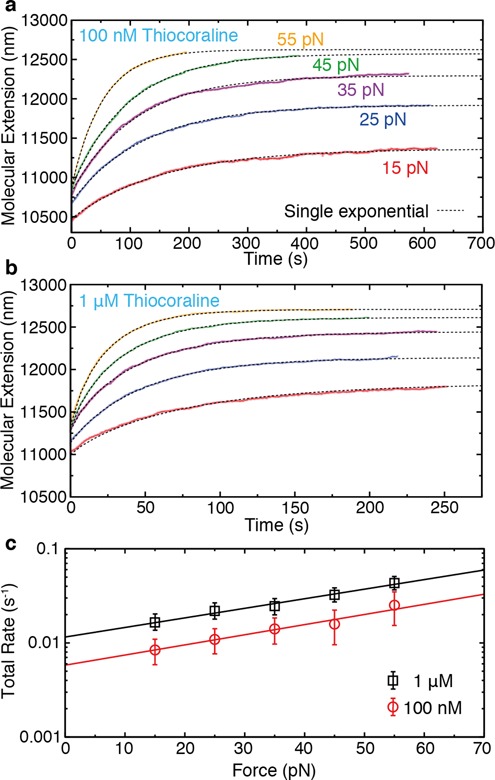
Total macroscopic kinetic rate (*k*_tot_) from force-jump experiments. (**a, b**) DNA extension as a function of time after increasing the force from 2 pN to a preset value of: 15 pN (red), 25 pN (blue), 35 pN (purple), 45 pN (green), 55 pN (yellow). Relaxations are well described by a single-exponential function and allow us to obtain *k*_tot_ at the two concentrations investigated: 100 nM in panel (a) and 1 μM in panel (b). (**c**) Force dependence of *k*_tot_ at the two investigated concentrations. The intercalation rate increases exponentially with force with similar slopes for both concentrations.

To characterize the remarkably slow kinetics of Thiocoraline we performed force-jump experiments at two different concentrations (Figure 3a and b). The relaxation of the molecular extension at each force allowed us to characterize the total kinetic rate of intercalation as a function of force. Relaxation curves are well described by single-exponential kinetics, with a total rate that increases exponentially with force at both concentrations (Figure 3c). For a bimolecular reaction where *k*_on_ is much larger than *k*_off_, the total rate (*k*_tot_ = *k*_on_ + *k*_off_) should be proportional to ligand concentration. However, the total kinetic rates observed at 1 μM were found to be only ∼2-fold faster than those obtained at 100 nM, whereas at 10 nM we observed an ∼10-fold decrease in relation to 100 nM, suggesting that binding is not a purely bimolecular process.

**Figure 4.**
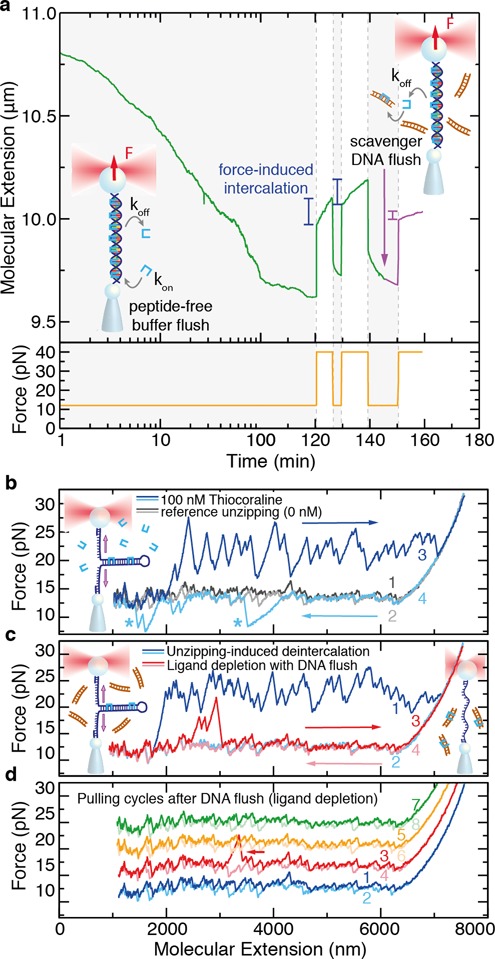
Wash-off experiments using scavenger DNA. (**a**) DNA extension as a function of time (green) in a wash-off experiment performed at 12 pN (orange) using peptide-free buffer. The extension decreases with time due to the unbinding of Thiocoraline. However, upon increasing the force after 2 h, an exponential elongation is observed, showing that small amounts of ligand remained in the microfluidics chamber. This effect can be suppressed by transiently flushing a high concentration of scavenger DNA (purple). Normal timescale after 100 min. (**b**) Unzipping the DNA hairpin (6.8 kb) without ligand shows a characteristic quasi-reversible sawtooth pattern due to base-pair disruption (gray). In the presence of Thiocoraline, a series of force peaks is observed during pulling (dark blue) indicative of binding events. During refolding (light blue), kinetic states that temporarily block the hairpin rezipping are sometimes observed (*). (**c**) To test the wash-off method, we flushed the scavenger DNA in, and removed the bound ligands by unzipping and rezipping the hairpin (blue). If the hairpin is immediately unzipped again (red), very few binding events remain. (**d**) Consecutive unzipping cycles performed during the following 10 min do not show binding (cycles shifted upward for clarity), and only occasionally (<20%) individual binding events are observed (arrow).

To verify this we directly measured the off-rate *k*_off_(*F*) from wash-off experiments. In these experiments, an intercalated DNA molecule is hold at a constant force in the optical tweezers setup, while the chamber is flushed with peptide-free buffer (Figure 4a). However, the low solubility and hydrophobicity of Thiocoraline makes difficult to fully remove the peptide from the chamber in such experiments. In order to create an effective depletion of ligands, we flushed scavenger DNA (see the Materials and Methods section). Thus, ligands that unbind from the tethered molecule or from the microfluidics chamber are sequestered and dragged away. For instance, in the experiment shown in Figure 4a, even after 2 h of peptide-free buffer flush, force-induced intercalation occurs if the force is increased to 40 pN. However, a brief flush of 10-μM bp DNA largely blocks this effect in a subsequent force-jump (Figure 4a, purple). This shows that ligand-free buffer flow is not sufficient to wash the chamber in constant-force experiments.

To test how reliable is this approach to generate ligand depletion, we performed unzipping experiments with a long DNA hairpin (6.8 kb). In DNA unzipping experiments the hairpin is pulled from the two ends and base pairs are sequentially disrupted (Figure 4b). In the absence of ligands, the unzipping shows a characteristic sawtooth pattern at a force ∼15 pN that is quasi-reversible at the experimental pulling speed (Figure 4b; dark gray is unzipping and light gray rezipping). In the presence of 100-nM Thiocoraline (Figure 4b, dark blue), the unzipping pattern shows a series of peaks at *F* ≥ 20 pN due to the increased DNA stability at the positions where bis-intercalators are bound. In these experiments, the molecule can be rezipped again by decreasing the distance between beads (Figure 4b, light blue), recovering the typical sawtooth pattern that indicates DNA hybridization. Interestingly, in the presence of Thiocoraline, a few specific locations of the hairpin (indicated with an *) require lower forces to refold. We attribute this rare events to the formation of competing non-native structures that are locally stabilized by Thiocoraline, and delay the formation of the native hairpin. The molecule can then be repeatedly pulled in the presence of 100-nM Thiocoraline, obtaining equivalent unzipping (dark blue) and rezipping (light blue) curves.

To test the DNA wash-off method, we flushed the scavenger solution while holding the intercalated DNA molecule partially unzipped. After a 3-min flush, the molecule is unzipped to remove all the intercalated peptide and rezipped again (Figure 4c, blue). In the following unzipping cycle, only three binding events are detected (Figure 4c, red). Successive pulling cycles are indistinguishable from naked DNA unzipping experiments (Figure 4d), and only individual binding events (e.g. red FEC in Figure 4d) are occasionally observed (<20%). This assay is therefore particularly well suited to detect subnanomolar concentrations of ligands, as even single-binding events give a clear footprint in the DNA unzipping pattern (i.e. over 6800 potential binding sites). We conducted similar experiments starting with a lower initial concentration of 10-nM Thiocoraline with equivalent results (Supplementary Figure S5). Consequently, by using scavenger DNA in the wash-off experiments, it is possible to create a depletion of ligand in the experimental area and, as shown below, perform accurate off-rate measurements at different forces even for an initially high concentration of ligand (100 nM).

### The kinetic off-rate (*k*_off_) reveals a binding intermediate

**Figure 5.**
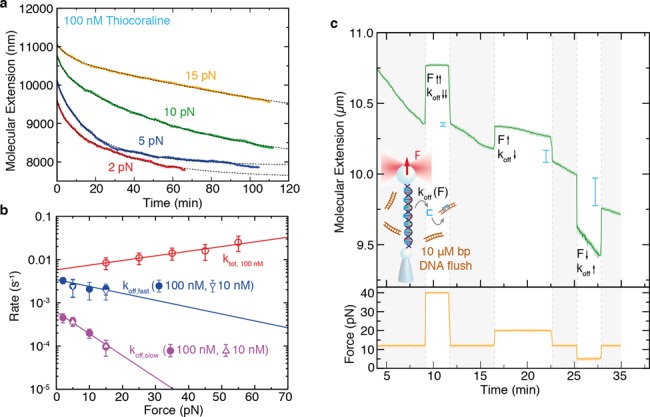
Off-rate kinetics of bis-intercalation. (**a**) DNA extension as a function of time in wash-off experiments starting at 100-nM Thiocoraline at four different forces, and fit to a double-exponential function (dotted gray). (**b**) Force dependence of the macroscopic kinetic rates: fast off-rate (blue), slow off-rate (purple). In red, *k*_tot_ from force-jump experiments is plotted as a reference. A fit to Equation (7) (solid lines) yields the macroscopic zero-force rates (*k*_off, slow_(0) = 6.2(7) · 10^−4^*s*^−1^, *k*_off, fast_(0) = 3.4(4) · 10^−3^*s*^−1^) and transition-state distances ($x^{\dagger }_{\text{off,slow}}=-0.48(5)$ nm, $x^{\dagger }_{\text{off,fast}}=-0.15(4)$ nm) for each process. Values are reported as mean ± SD, *N* ≥ 3. (**c**) Wash-off experiment in which different forces (yellow) are sequentially tested within the same kinetic trace. The molecular extension (green) is initially followed at a constant force of 12 pN. The slope of the molecular extension is proportional to the number of bis-intercalators that unbind from DNA per unit time and hence it is indicative of how the off-rate (*k*_off_) changes with force. Increasing the force from 12 pN (shadowed in gray) to a higher force value (40 pN, 25 pN) causes a decrease on the slope and hence in *k*_off_, whereas decreasing the force to 5 pN causes the opposite effect. We also indicated the measured change in molecular extension (blue lines) when the force is set again at 12 pN after a few minutes at 40 pN, 25 pN and 5 pN, showing that ligands unbind faster as force is decreased.

We investigated the off-rate kinetics of Thiocoraline at different forces in the DNA stretching setup. We first followed the unbinding reaction in DNA molecules equilibrated at 100-nM Thiocoraline, where the initial binding density is large (Figure 5a). The off-rate kinetics are remarkably slow at all forces (in the order of hours), showing a strong force-dependence. A single-exponential function fails to fit the experimental data (Supplementary Figure S6a), but experiments are well described by a double-exponential relaxation (Figure 5b) whose amplitude *A* is dominated by the slower off-rate process (*A*_slow_ > 75%, for all forces). To verify that the observed off-rates (*k*_off, fast_ and *k*_off, slow_) are independent of the initial binding fraction, we repeated measurements with DNA molecules equilibrated at a much lower Thiocoraline concentration (10 nM; Supplementary Figure S6b), finding rates that are compatible with the ones obtained at 100 nM (Figure 5b). The strong force dependence of the unbinding rate can be easily visualized by changing the applied force in the course of a single experiment (Figure 5c), allowing to slow down (speed up) the unbinding reaction as force is increased (decreased).

**Figure 6.**
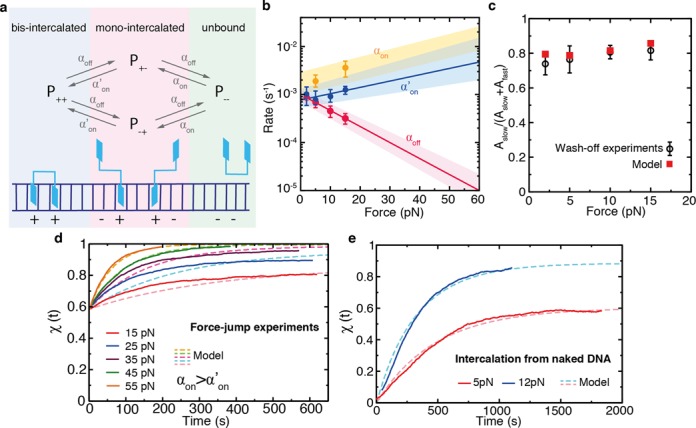
A kinetic three-state model with microscopic rates $\alpha _{\text{on}}, \alpha _{\text{on}}^{\prime }$ and α_off_. (**a**) Scheme of the kinetic model representing the different accessible states: unbound ligand (green), fully bis-intercalated (blue) and mono-intercalated intermediate (red). The binding of the first intercalating moiety is bimolecular (with a rate α_on_ proportional to ligand concentration), whereas the transition from the mono-intercalated intermediate to a fully bis-intercalated state is unimolecular (with rate $\alpha _{\text{on}}^{\prime }$). On the other hand, the removal of any of the two intercalative moieties is driven by a single off-rate α_off_. (**b**) Microscopic rates $\alpha _{\text{on}}^{\prime }$, α_on_ and α_off_ that best describe the experiments. The values of $\alpha _{\text{on}}^{\prime }$ (blue points) and α_off_ (red points) are determined from the macroscopic rates measured in wash-off experiments (Figure 5b). A fit to Equation (7) (red and blue lines) is used to extrapolate the high force values and their associated uncertainty range (shaded area). The rates α_on_ are determined from intercalation experiments with naked DNA (panel (e) and Supplementary Section S5). (**c**) Ratio of amplitudes of the fast and slow macroscopic off-rates measured in the wash-off experiments (black) compared with the model prediction (red). (**d**) Fractional elongation of the force-jump experiments from Figure 3a (solid lines) and comparison to the kinetics predicted by the model (dashed lines). (**e**) Fractional elongation of intercalation experiments (100-nM Thiocoraline) starting from a naked DNA molecule at 5 and 15 pN (solid lines) and comparison to the kinetics predicted by the model (dashed lines).

What is the origin of the two timescales observed in the wash-off experiments? The presence of two off-rates, together with the previous observation of an on-rate that is not proportional to ligand concentration, can be rationalized by considering the existence of an intermediate state in the binding reaction. A minimal model in which the binding of Thiocoraline proceeds through a mono-intercalated intermediate state (Figure 6a) and that considers three microscopic kinetic rates (α_on_, $\alpha _{\text{on}}^{\prime }$ and α_off_) is sufficient to reconcile all observations. The model can be analytically solved (Supplementary Section S5), and the values of $\alpha _{\text{on}}^{\prime }(F)$ and α_off_(*F*) directly determined from the macroscopic rates *k*_off, fast_(*F*) and *k*_off, slow_(*F*) measured in wash-off experiments (Figure 6b). The microscopic kinetic rates depend exponentially with the applied force *F* and can be fit to the expression

(7) \begin{equation*} \alpha _{\text{on'}/\text{off}}(F)=\alpha _{\text{on'}/\text{off}}(0)\exp (Fx^{\dagger }_{\text{on'}/\text{off}}/\text{k}_{\text{B}}T), \end{equation*}

where *k*_B_ is the Boltzmann constant and *T* is the temperature. Using the microscopic rates determined in this way, the master equations can be solved to reproduce three types of experiments with different initial conditions: (i) wash-off experiments, (ii) force-jump experiments and (iii) intercalation kinetics starting from naked DNA. Remarkably enough, the amplitudes (*A*_slow_ and *A*_fast_) observed in the wash-off experiments are in excellent agreement with the amplitudes predicted by the model if the intermediate state is assumed to have an extension 0.4 times that of a bis-intercalated state (Figure 6c), supporting the hypothesis that the intermediate state is mono-intercalated (with an elongation ∼50% that of the bis-intercalated state which is 0.68 nm). Similarly, the fractional elongation χ(*t*) (0 for naked DNA, and 1 for fully bis-intercalated DNA) can be calculated from the molecular extension *x*(*t*) of kinetic experiments (see Supplementary Section S5) and compared to the prediction of the model. The model describes well the equilibrium extensions and kinetics of the force-jump experiments (Figure 6d), and of constant-force experiments that start with a naked DNA molecule (Figure 6e). Overall, this three-state model captures the essential features of the experiments providing a mechanistic explanation for the binding kinetics of Thiocoraline.

The observed increase of $\alpha ^{\prime }_{\text{on}}$ with force is characterized by a distance $x^{\dagger }_{\text{on'}}=0.12(5)$ nm, whereas α_off_ steeply decreases with force ($x^{\dagger }_{\text{off}}=-0.29(2)$ nm). An off-rate that decreases with force is in striking contrast with previous studies of the mono-intercalator Actinomycin (19) and a threading intercalator (21), where no intermediate was observed. Whereas force facilitates both the binding and unbinding of these ligands due to base-pair destabilization (19,21), this is not the case for Thiocoraline. Our data show that DNA stretching kinetically stabilizes the bound state, suggesting that bis-intercalators behave like a molecular ‘wedge’ (i.e. applying force clamps the ligand, whereas compression pushes the intercalator out). According to our model, the characteristic elongation of each intercalating moiety can be inferred from the transition-state distances ($x^{\dagger }_{\text{on'}}$ and $x^{\dagger }_{\text{off}}$). For the transition between the intermediate state and the fully bound state, this yields a value ($\Delta x_{\text{eq}}=x^{\dagger }_{\text{on'}}-x^{\dagger }_{\text{off}}=0.41(8)$ nm). This is in good agreement with the expected distance change for this transition ∼0.4 nm (equal to the additional 60% of elongation to reach a full bis-intercalated state). From the value of α_on_ we can also infer the rate of association at zero force ($k_a=\frac{\alpha _{\text{on}}(0)}{[\text{Thio}]}=1.7(3)\cdot 10^{4}$ *M*^−1^*s*^−1^). This rate is slow if compared to mono-intercalators or charged bis-intercalators (YOYO-1) (41,42), but is in good agreement with other structurally similar non-charged bis-intercalators (43,44). Finally, the presence of a binding intermediate reproduces well the titration curve for the DNA contour length (Figure 2c).

### Single-molecule footprinting of preferred binding sites

To determine the preferred binding sites of Thiocoraline, we developed a single-molecule footprinting assay based on DNA unzipping. Unzipping-based methods have proven useful to locate binding of nucleosomes and restriction enzymes with base-pair resolution (45,46). However, to the best of our knowledge, this is the first time that they are used to directly determine the binding site of small ligands.

**Figure 7.**
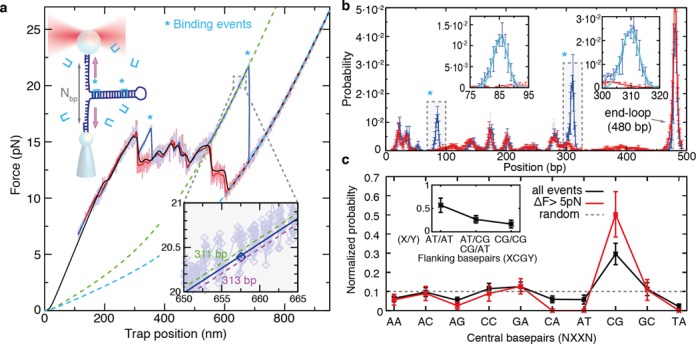
Single-molecule footprinting reveals preference for CpG steps. (**a**) DNA unzipping (blue) and rezipping (red) of a 480-bp DNA hairpin in the presence of 5-nM Thiocoraline. Pulling speed is 70 nm/s and data are recorded at 1 kHz (light colors) and filtered to 10 Hz (dark colors). The binding of a ligand brings an extra stabilization energy, causing a force peak in the unzipping curve (*). A theoretical prediction of the equilibrium FDC is shown in black and used for alignment. An FJC curve corresponding to a fully unzipped hairpin (*n* = 480 bp, cyan dashed) and one that passes close to a binding event (*n* = 311 bp, green dashed) are plotted as a reference. Inset: for each datapoint of the unzipping/rezipping curves we found the most probable number of open base pairs *n*, by finding the theoretical curve that passes closest to it. For the highlighted datapoint (blue), the distance is minimized for *n* = 312 bp (blue line). (**b**) Histogram of *n* values of the unzipping curve (blue) and rezipping curve (red) from panel (a). The histograms match each other except for the positions in which there is a binding event (highlighted in gray, *). Insets: zoom of a binding peak and fit to a Gaussian function (cyan). (**c**) Normalized probability of clamping sites XX: including all binding events (black), or by selecting events with rupture forces 5 pN higher than the average unzipping force (red). The dashed line indicates the distribution expected for a ligand that uniformly binds all sites. Inset: normalized probability of observing A-T bp or C-G bp flanking the preferred binding site XCGY. (*N* = 142 binding events, 104 unzipping curves and ∼1 binding event per cycle).

We repeatedly unzipped a 480-bp DNA hairpin in the presence of 5-nM Thiocoraline (Figure 7a). In the absence of ligand the unzipping and rezipping curves fully overlap (Supplementary Figure S8a, top). However, in the presence of ligand, the unzipping curves show a series of force peaks indicative of individual binding events (Figure 7a, marked with *). Additional pulling curves are shown in Supplementary Figure S8, where binding events to other positions along the hairpin are observed. Experiments are typically performed at a concentration where a few binding events per pulling cycle are detected. This is done to determine the position of the peaks accurately and to ensure that binding events are tested homogeneously along the DNA fragment. The overall footprinting map of the molecule and its sequence selectivity is recovered by collecting a large number of pulling cycles (Supplementary Figure S9) and correlating the position of the binding events to the DNA hairpin sequence.

First, the position of the binding events was determined from a statistical analysis of the FDCs (see the Materials and Methods section) (28,47). Briefly, the experimental FDCs were aligned to the theoretical prediction based on the nearest-neighbor model (Figure 7a, black) (33). For each experimental datapoint we then determined the most probable number of unzipped base pairs *n*, by finding the elastic curve (see the Materials and Methods section) that passes closest to the datapoint (Figure 7a, inset) (28,37,47). In this way, we assigned a number of open base pairs *n* to each experimental datapoint (Figure 7b). FDCs are reversible at this pulling speed, and consequently the unzipping and rezipping histograms (Figure 7b, red and blue, respectively) overlap except for those positions in which there is a binding event (Figure 7b and Supplementary Figure S8b, blue peaks). By fitting these extra peaks to Gaussian distributions the position of each binding event could be identified (Figure 7b, insets). In this way, we determined the probability of binding for each different dinucleotide pair (Figure 7c, black). Remarkably, we find that Thiocoraline bis-intercalates all 10 possible dinucleotides, but shows a clear preference to clamp CpG steps. Moreover, by analyzing the neighboring bases we also find that flanking A-T base pairs are favored (i.e. ACGT, TCGA, ACGA/TCGT) (Figure 7c, inset). The sensitivity of the technique can be increased by only selecting events that show rupture forces greater than a given threshold (e.g. 5 pN) above the local unzipping force. As expected, this analysis shows a higher peak at the specific binding sites (Figure 7c, red), confirming the selectivity of Thiocoraline for clamping CpG steps. An estimation of the measurement error can be found by considering alternative distances between the position of the binding peaks and the clamping positions (Supplementary Figure S10).

So far, the existence of specific binding sites for Thiocoraline was rather controversial. Although the peptide showed a general preference for G-C-rich regions, protected sites were not observed in classical DNA footprinting experiments, suggesting that it had little sequence selectivity (31). On the other hand, fluorescent melting experiments with short oligonucleotides showed a clear preference for G-C-rich oligos and particularly those containing CpG steps (32). Our direct measurement of binding positions from unzipping experiments demonstrates that Thiocoraline has a specific affinity for CpG steps (particularly those flanked by A-T base pairs), although it can also clamp a large number of alternative dinucleotide steps, in agreement with our stretching results that show binding saturation at high enough concentrations (Figure 2c). Moreover, this binding pattern seems remarkably similar to that exhibited by the quinoxaline bis-intercalator Echinomycin that shares several structural similarities with Thiocoraline that might confer specificity for these motifs (5,48–49). In addition, information on cooperative binding should also be accessible with this technique. The presence of cooperative effects between contiguous binding sites is expected to show both: (i) a higher frequency of binding events at these positions and (ii) increased rupture forces than in non-cooperative sites. In fact, the DNA fragment used in these experiments contains a motif (TCGTACGA) that corresponds to two juxtaposed specific binding sites. Remarkably, binding events of Thiocoraline at this position are particularly abundant and show high rupture forces, suggesting cooperative binding. Although more experiments are needed to confirm this result, general synergistic effects should be detectable with our technique.

Overall, our footprinting assay gives direct access to individual binding events of ligands due to the higher thermodynamic stability that they induce upon binding DNA. Consequently, specific and non-specific binding sites are observed in a single experiment, without relying on indirect measurements such as differential access of cleavage agents, shifts in gel-mobility (difficult for small ligands) and/or radiolabeling (24–26). Similarly, DNA templates can be much longer than typical gel footprinting assays, and a statistically significant amount of data can be collected in a few hours. We therefore anticipate that this method may be widely applicable to determine the sequence specificity of a large number of small ligands that are difficult to characterize with traditional assays.

## DISCUSSION

Thiocoraline, an anticancer peptide that bis-intercalates DNA, exhibits an unusual slow kinetics of binding. We have exploited this feature to clarify the mechanical properties of bis-intercalated DNA in the low- and high-force stretching regimes (16–17,23,40). Contrary to previous studies in which equilibrium FECs were used, we have worked with reversible metastable FECs in which the fraction of ligand is kept constant. We found that the bending rigidity of DNA remains unchanged, which we interpret in terms of a compensation between the electrostatic effect of unwinding the DNA double helix (larger interphosphate distance) and the straightening induced by base-stacking interactions with the intercalated chromophores.

We also showed that Thiocoraline has a high binding affinity to DNA finding compatible values from equilibrium and kinetic measurements (*K*_*d*_ = 77 ± 13 nM) and reaching the nearest-neighbor exclusion limit of *n* = 4 at submicromolar concentration (50). Remarkably enough, the affinity to DNA is dominated by a very slow macroscopic off-rate (*k*_off_(0) = 6.2(7) · 10^−4^ *s*^−1^) that steeply decreases with force. This allows us to kinetically control ligand unbinding by changing the applied force, obtaining extremely long-lived complexes at even moderate stretching forces (*F* < 20 pN). From a structural point of view, this indicates that a pulling force further accommodates the bound ligand, stabilizing the bis-intercalated tetranucleotide motif. This might be common to other cytotoxic DNA bis-intercalators (e.g. Echinomycin, Triostin), and we hypothesize it is very likely related to the high toxicity of this family of anticancer drugs that show activity at concentrations much below their *K*_*d*_ values (e.g. Thiocoraline IC_50_ ∼ 200 pM (31)). To accurately determine these unbinding rates, we have used a combination of stretching and unzipping experiments that allowed us to ensure that subnanomolar traces of bis-intercalator did not remain in the wash-off experiments. This methodology might be particularly relevant in future studies of other highly hydrophobic and low solubility ligands (28). Our experiments also reveal that the binding pathway proceeds through a long-lived mono-intercalated intermediate (lifetime ∼10 min) which elongates DNA by 0.28 nm (which is 0.4 times the elongation of a fully bis-intercalated ligand, 0.68 nm) contributing to the long lifetime of the peptide (hours). The stretching-unzipping methodology allows us to extract the force dependence of the microscopic rates (α_on_(*F*), $\alpha _{\text{on}}^{\prime }(F)$ and α_off_(*F*)) from the characteristic timescales of kinetic experiments performed with varying conditions.

To determine the sequence selectivity of the ligand, we developed a novel DNA unzipping assay to perform single molecule DNA footprinting experiments. Using this assay we could directly locate single-binding events finding that Thiocoraline shows a higher preference to clamp CpG steps, particularly those flanked by A-T base pairs. We foresee that this assay might be of extreme utility to determine the binding site of small ligands as it gives direct access to thermodynamically stabilized sites without requirement of cleavage enzymes and radiolabeling techniques, allowing to rapidly recover the binding sites in DNA fragments 10-fold longer than typical footprinting templates (25,26).

So far, Thiocoraline has been observed to interfere with primer extension by DNA polymerase α, showing a very prolonged inhibition of DNA replication even after 48-h removal of the drug (51), in contrast with the typical topoisomerase inhibition mechanism of most DNA intercalators (e.g. doxorubicin). However conclusive studies are lacking, being unclear how its selectivity for cancer cells arises (49,51–52). In view of these results, we suggest that the increased binding stability of the ligand at moderate stretching forces might create long-lived kinetic roadblocks that stall the progression of replication and transcription enzymes at picomolar drug concentrations. Moreover, we have shown that these roadblocks might also happen through the stabilization of non-native kinetic structures that are particularly present in DNA-melted regions during transcription and replication. More importantly, the determined sequence specificity suggests that Thiocoraline might accumulate at specific genomic locations rich in CpG steps. Notably, CpG sites are sparsely distributed in human genomes, accumulating in many regulatory regions and having much lower frequencies elsewhere (53). We hypothesize that this inhomogeneous distribution of CpG islands might be related to the selectivity of Thiocoraline for carcinogenic cell lines (51).

## SUPPLEMENTARY DATA

Supplementary Data are available at NAR Online.

SUPPLEMENTARY DATA
