# Supplementary material for: Single-molecule kinetics and footprinting of DNA bis-intercalation: the paradigmatic case of Thiocoraline
Source: Nucleic Acids Res. 2015 Feb 17;43(5):2767–79. doi: 10.1093/nar/gkv087 (PMC4357703; doi:10.1093/nar/gkv087)
Supplement: SUPPLEMENTARY DATA [file supp_43_5_2767__index.html]

Single-molecule kinetics and footprinting of DNA bis-intercalation: the paradigmatic case of Thiocoraline — SUPPLEMENTARY DATA 

# Single-molecule kinetics and footprinting of DNA bis-intercalation: the paradigmatic case of Thiocoraline

## SUPPLEMENTARY DATA

**Files in this Data Supplement:**

- Supplementary Information
- Movie Clip
