# Supplementary material for: Single-molecule kinetics and footprinting of DNA bis-intercalation: the paradigmatic case of Thiocoraline
Source: Nucleic Acids Res. 2015 Feb 17;43(5):2767–79. doi: 10.1093/nar/gkv087 (PMC4357703; doi:10.1093/nar/gkv087)
Supplement: SUPPLEMENTARY DATA [file supp_gkv087_nar-03685-f-2014-File014.pdf]

Supplementary Information for: Single-molecule kinetics and footprinting of DNA bis-intercalation: the paradigmatic case of Thiocoraline

J. Camunas-Soler,[1,2] M. Manosas,[1,2] S. Frutos,[1,2] J. Tulla-Puche,[3,2] F. Albericio,[3,2] and F. Ritort[1,2,*]

[1]*Small Biosystems Lab, Departament de Física Fonamental, Facultat de Física, Universitat de Barcelona, Barcelona, Spain*

[2]*CIBER de Bioingeniería, Biomateriales y Nanomedicina, Instituto de Salud Carlos III, Madrid, Spain*

[3]*Institute for Research in Biomedicine(IRB Barcelona), Barcelona Science Park, Baldiri Reixac 10-12,08028, Barcelona, Spain*

[*] fritort@gmail.com

CONTENTS

S1. Force-jump experiments 3

S2. DNA stretching experiments in the overstretching region 5

S3. Elastic properties of bis-intercalated DNA 7

S4. Wash-off experiments and off-rate kinetics 8

S5. Three-state kinetic model 10

S5.1. Determining the microscopic rates α'_{on} and α_{off} from wash-off experiments 11

S5.2. Comparing the values of the amplitude as predicted by the model with the results from wash-off experiments. 11

S5.3. Reproducibility of force-jump experiments and intercalative experiments starting from naked DNA ($\alpha_{\text{on}} \neq 0$): 13

S6. Single-molecule footprinting of preferred binding sites 15

References 18

S1. Force-jump experiments

We performed force-jump experiments to recover the equilibrium FEC of Thiocoraline-DNA and to measure the kinetic rates of binding. The experiments were performed in two ways: (i) bidirectional stepwise force-jumps, and (ii) unidirectional force-jumps from low force. In the first type of experiments (Figure S1**a**), the force was increased stepwise every ∼5 min up to a maximum preset force. Then the protocol was reversed and the force stepwise decreased down to the initial value. By doing so, we obtained a forward and backward relaxation curve for each force. The relaxations were well described by a single-exponential function, from which we obtained the forward and backward asymptotic extensions (Figure S1**b**). The equilibrium extension at each force $x_{eq}^{[Thio]}(F)$ was estimated as the average of both values. To show that $x_{eq}^{[Thio]}(F)$ does not depend on the stretching history, we independently measured this value from unidirectional force jumps (Figure S1**c**). In these experiments, the force was increased from 2 pN to a preset value, and the relaxation of the molecular extension was followed until reaching saturation (∼ 10 min). Consecutive force-jumps between 2 pN and the different preset forces were performed in this way, and fitted to a single-exponential function (Figure S1**d**). The $x_{eq}^{[Thio]}(F)$ values obtained with both methods were fully compatible. From this alternative measurement, we also obtained the total kinetic rate $k_{\text{tot}}(F)$ at each force (Figure 3, main text). All force jumps were performed with a force-feedback operating at 1 kHz in which force was changed in less than 0.1 s and maintained constant to the preset value.

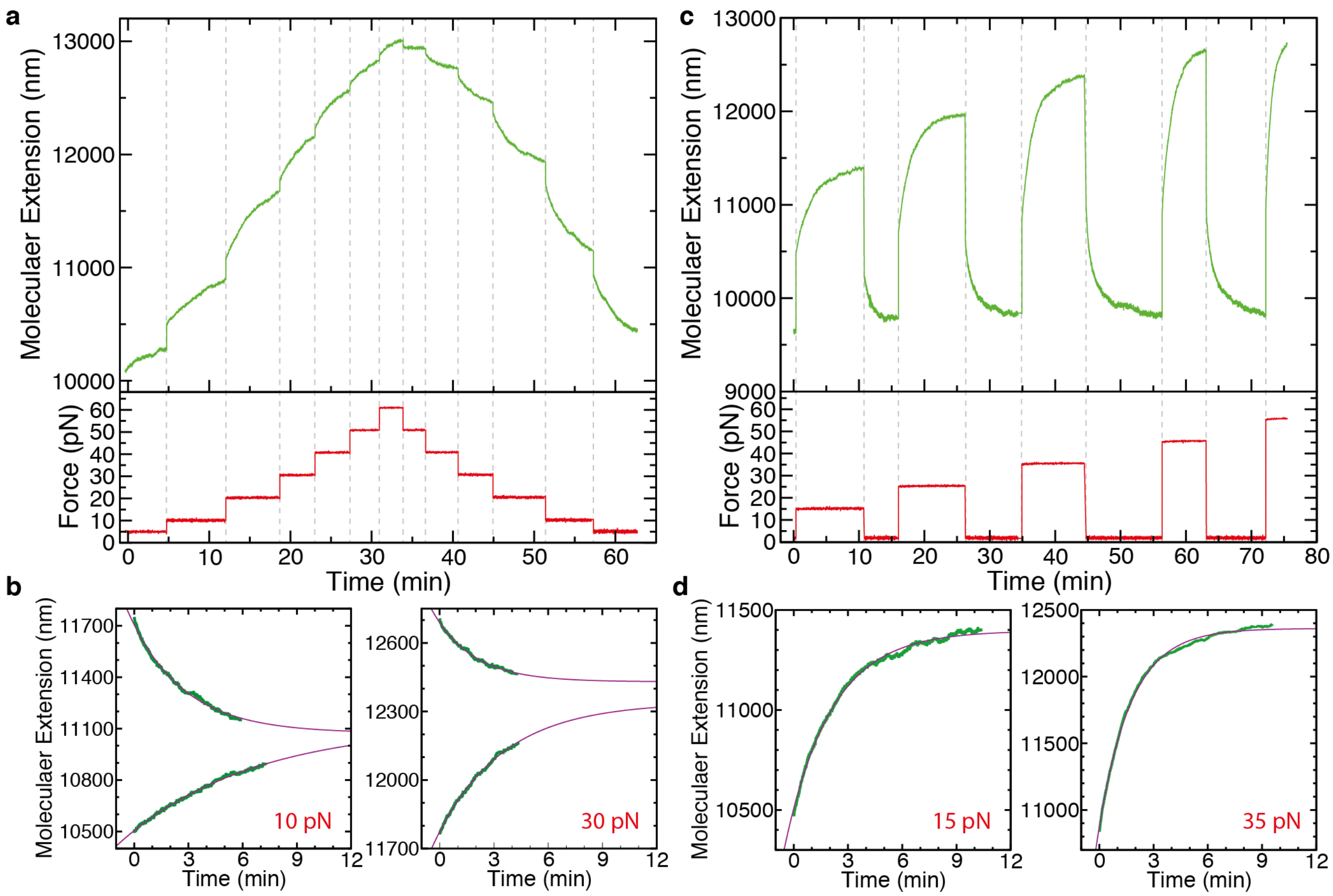

Figure S1. **Force-jump experiments.** **(a)** Typical bidirectional force-jump experiment. The force (red) is increased stepwise up to a maximum value using force-feedback and then decreased down to the initial value using a reversed protocol. In this way, the relaxation of the molecular extension (green) is obtained in the forward and backward direction for different force values. **(b)** Relaxation of the molecular extension (green) for two forces from panel (a). Data is fitted to a single exponential function (purple) from which the equilibrium extension $x_{\rm eq}^{\rm [Thio]}(F)$ at each force is determined. **(c)** Typical unidirectional force-jump experiment. The force (red) is increased from 2 pN to a preset value, and the relaxation of the molecular extension (green) is measured until reaching saturation. Then the force is decreased again to 2 pN and the measurement repeated for a set of different forces. **(d)** Relaxation of the molecular extension (green) at two forces from panel (c). Data is fitted to a single exponential function (purple), obtaining $x_{\rm eq}^{\rm [Thio]}(F)$ and the total kinetic rate $k_{\rm tot}$ at each force. Experiments correspond to 100 nM Thiocoraline.

.

S2. DNA stretching experiments in the overstretching region

The DNA pulling curves presented in the main text are performed up to a maximum stretching force of 40 pN. This force is below the overstretching transition (F_{OS} ∼63 pN at 100 mM NaCl), and allows us to obtain metastable FECs at the highest pulling speed of 1.5 μm/s for all Thiocoraline concentrations ([Thio]≤1 μM). However, if the maximum pulling force is increased to 70 pN, FECs show longer molecular extensions and larger hysteresis as compared to 40 pN, due to the greater intercalation achieved at the highest forces (Figure S2**a**). In fact, at 100 nM (concentration used for the experiments shown in the main text), hysteresis is observed also at the fastest pulling speed of 1.5 μm/s (Figure S2**b**), and we cannot pull fast enough to reach the metastable regime at this higher force. As previously observed for other intercalators [*S1*, *S2*], a tilted plateau related to the overstretching transition is observed. If experiments are performed at lower bis-intercalator concentrations, the equilibration time of the binding reaction τ_{eq} increases and the metastable FECs can be obtained again at the fastest pulling speed (Figure S2**d-f**, red). As expected, when we decrease ligand concentration the molecular extension becomes shorter (due to the lower fraction of ligand bound at low forces), and the slope of the force plateau decreases approaching the overstretching curve of naked dsDNA. These findings are in agreement with a mechanism in which the bis-intercalator binds to B-DNA, elongating and unwinding the double helix, and increasing the energy required to force-melt or fully unwind the bis-intercalated DNA (converting B-DNA into S-DNA). At concentrations below 10 nM, fast pulling experiments show a molecular extension slightly longer (<5%) than naked dsDNA and an overstretching plateau that is slightly tilted, indicative of a few bound intercalators (Figure S2**e, f**). Remarkably, at this low concentrations slower pulling rates show negative hysteresis along the overstretching region (the force is higher in the releasing as compared to the stretching part of the pulling cycle, Figure S2**d-f**). These results suggest that Thiocoraline is able to bind either force-melted regions or S-DNA, stabilizing the DNA double helix and therefore reducing the molecular extension at forces above the overstretching transition. In these experiments, once the force is reduced below the overstretching transition, the molecular extension remains longer than during stretching, confirming that Thiocoraline has bis-intercalated DNA at forces above the overstretching plateau. This is particularly visible in the 3 nM experiment (Figure S2**e**). In this condition, the stretch part of the cycle for the fast (dark red)

and slow (dark blue) pulling rate are very similar below the overstretching region. However, during releasing at the slow pulling rate (light blue) the apparent molecular extension is ∼700 nm longer than that observed at a fast pulling rate (light red), suggesting that most of the binding has happened at $F \geq F_{\mathrm{OS}}$. Recent evidence that force-melted DNA and S-DNA coexist at physiological conditions, and their similar extension, makes difficult to unambiguously attribute binding to any of both [*S3*, *S4*].

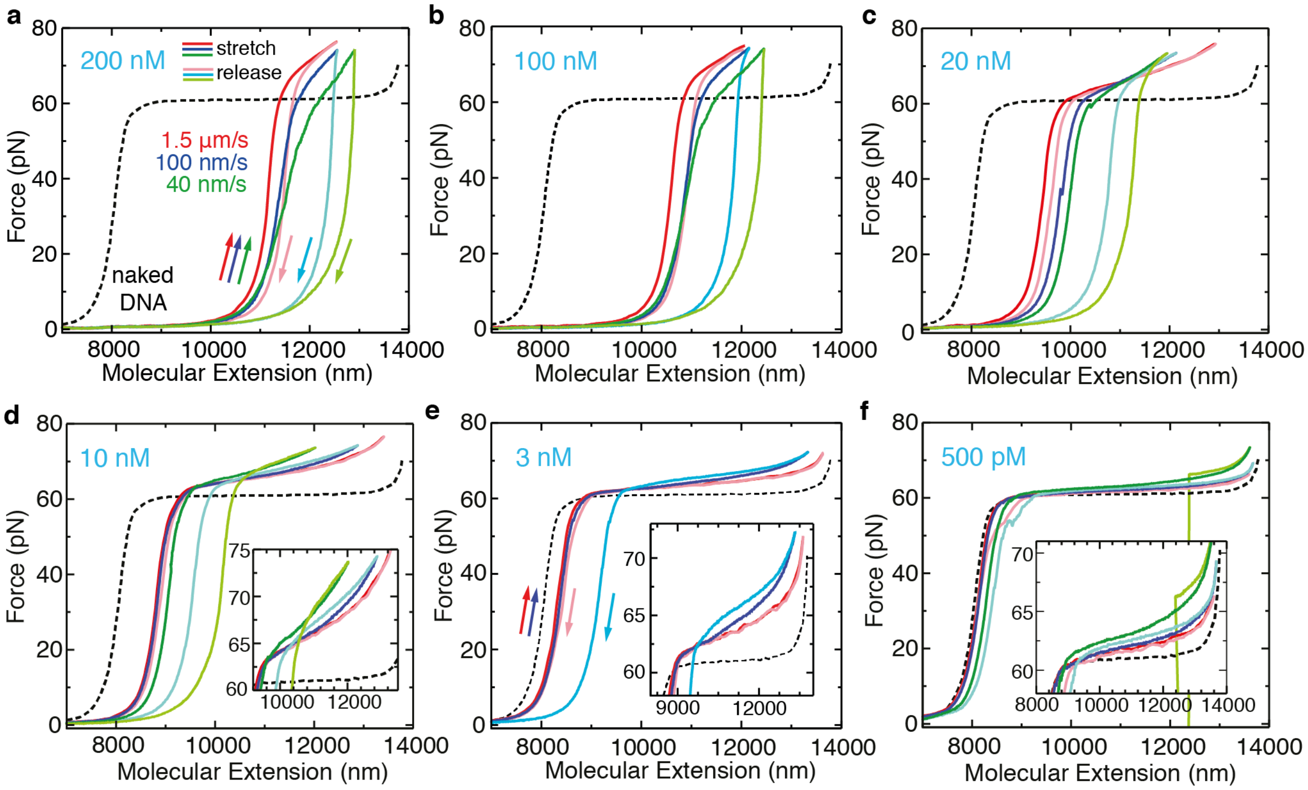

Figure S2. **Hysteresis in Thiocoraline pulling experiments.** FECs of DNA at six different Thiocoraline concentrations: 200 nM (a), 100 nM (b), 20 nM (c), 10 nM (d), 3 nM (e), 500 pM (f). Experiments are performed up to a maximum force of 70 pN at three different pulling speeds: 1.5 $\mu m/s$ (red), 100 nm/s (blue) and 40 nm/s (green), and dark and light colors correspond to the stretching and release part of the pulling cycle respectively. A FEC of naked dsDNA is shown as a reference (dotted black).The sudden force drop to 0 pN in the 40 nm/s (green) experiment in panel (f) corresponds to the breakage of the bead-DNA attachment.

S3. Elastic properties of bis-intercalated DNA

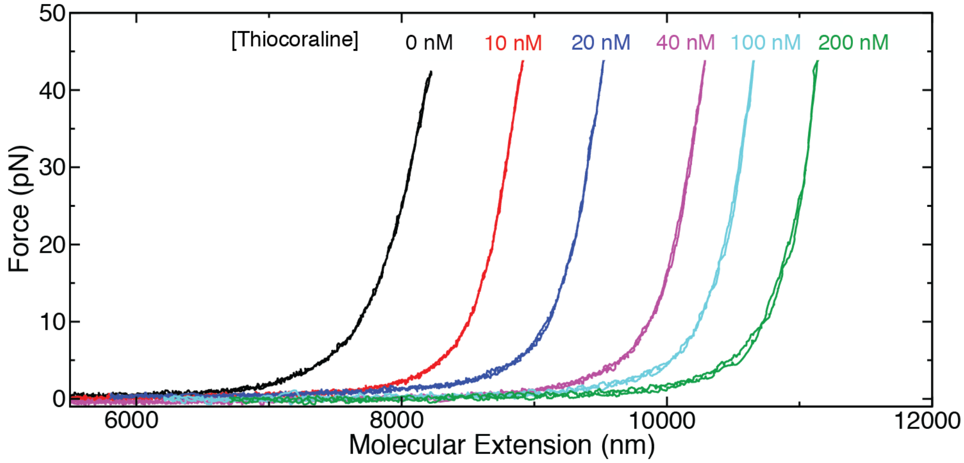

Figure S3. **Metastable FECs.** Metastable FECs at different Thiocoraline concentrations: 10 nM (red), 20 nM (blue), 40 nM (purple), 100 nM (cyan) and 200 nM (green). A FEC of naked dsDNA is shown in black as a reference.

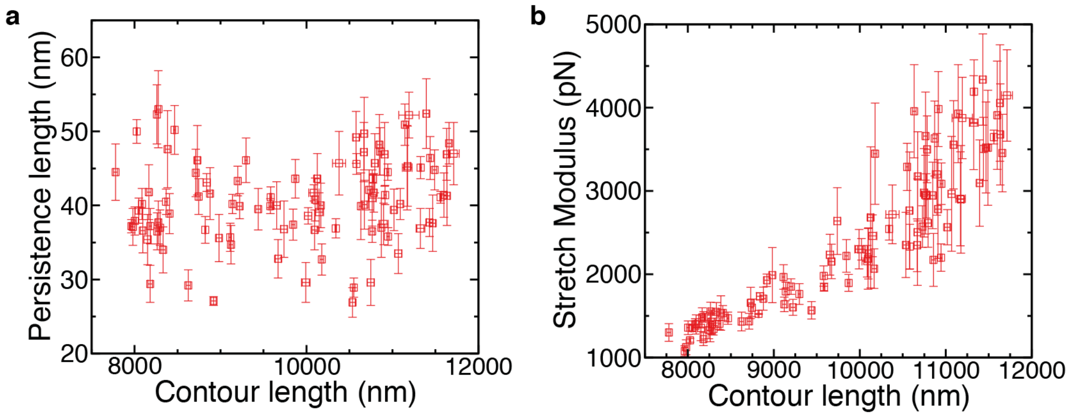

Figure S4. **Elastic parameters as a function of contour length.** (**a**) Persistence length (L_p) as a function of contour length. L_p is independent of the contour length of the bis-intercalated DNA molecule. (**b**) Stretch modulus (S) as a function of contour length. S monotonically increases with contour length, demonstrating that S is directly related to the fraction of bis-intercalator bound to the DNA molecule. The elastic parameters (L_p, l_0, S) are obtained by fitting the FECs of individual molecules to the WLC model (Eq. 2, main text). A set of consecutive pulling cycles for each molecule are used ($N > 5$), and results are shown as mean± SD. Experiments are performed at varying concentrations between 0.5-1000 nM Thiocoraline.

S4. Wash-off experiments and off-rate kinetics

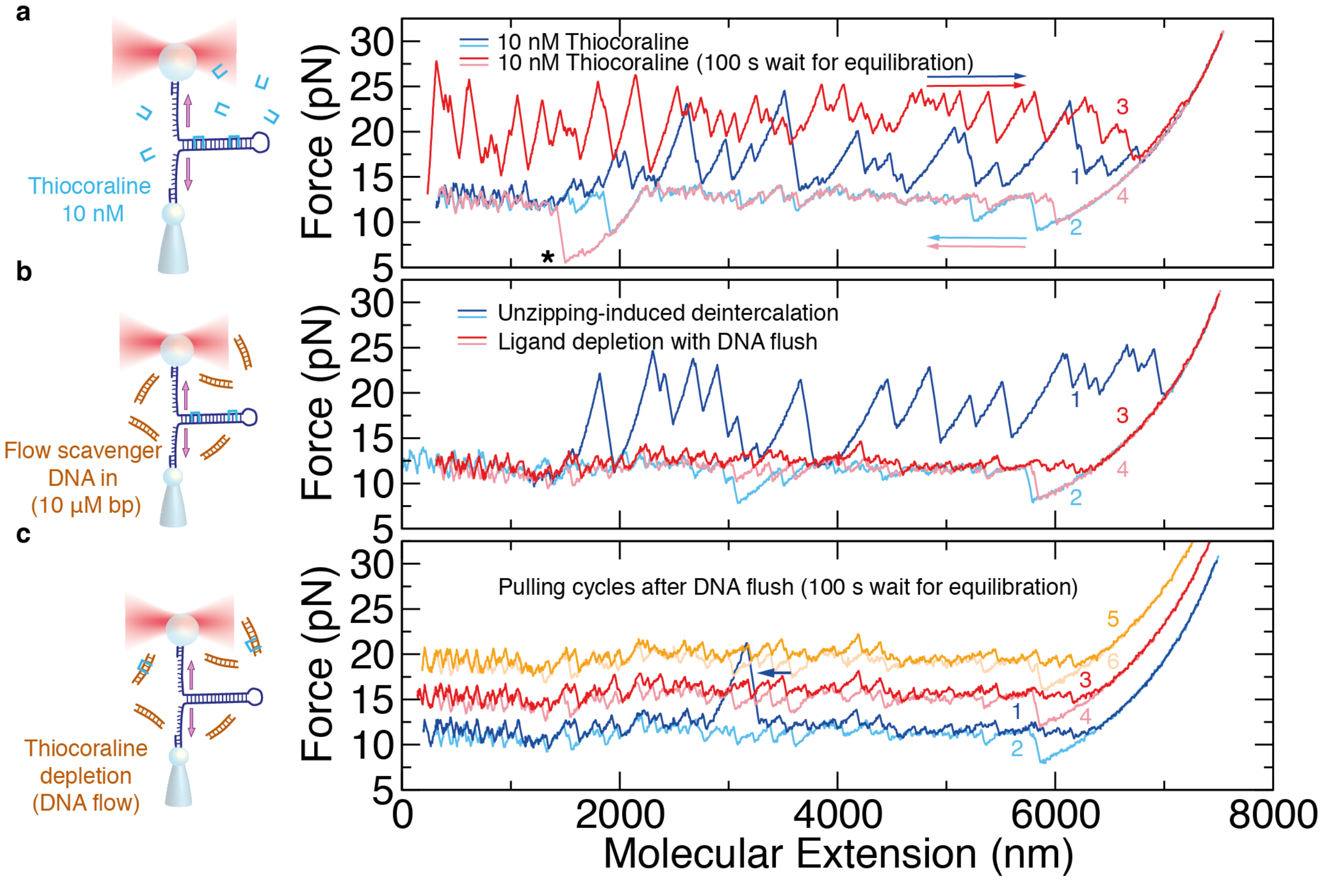

Figure S5. **DNA unzipping at 10 nM Thiocoraline and wash-off with scavenger DNA.** (**a**) Unzipping of the 6.8-kb DNA hairpin at 10 nM Thiocoraline (dark blue) shows a lower fraction of binding events than at 100 nM (Figure 4**b** in main text, blue) as expected. The number of bound intercalators can be kinetically increased by waiting 100 s between consecutive pulling cycles (dark red). Interestingly, at this much lower concentration the kinetic stabilization of non-native structures at specific locations (*) is also observed in the rezipping curves (light red and blue). (**b**) The bound ligands are removed by unzipping (dark blue) and rezipping (light blue) the hairpin, after flowing the scavenger DNA solution for 3 min. If the hairpin is immediately unzipped again (red), we do not observe any binding event showing that the experimental area is free of ligand. (**c**) Consecutive unzipping cycles performed during the following 15 min are indistinguishable from naked DNA (cycles are shifted upwards for clarity) and only very occasionally individual binding events (blue arrow) are observed. Pulling speed is 500 nm/s.

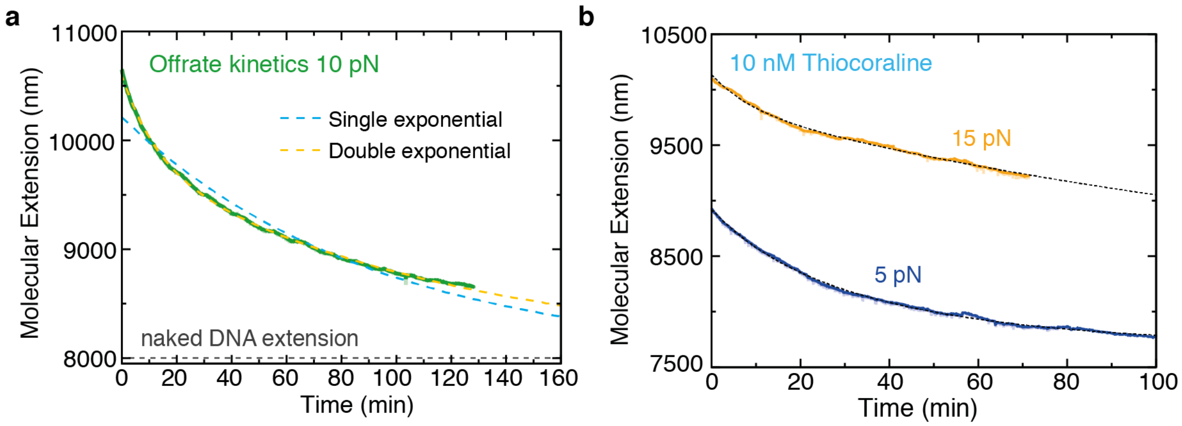

Figure S6. **Off-rate kinetics and fit to single and double exponential functions.** (**a**) Molecular extension as a function of time (green) in a wash-off experiment performed at 10 pN (initial Thiocoraline concentration was 100 nM). A double exponential function (yellow) fits well the data, whereas a single-exponential function (blue) does not. A reference of the extension of a naked dsDNA molecule at 10 pN is indicated in gray. (**b**) DNA extension as a function of time in wash-off experiments starting at a lower concentration of 10 nM Thiocoraline (note the initially shorter molecular extensions as compared to Figure 5**a** in the main text). Experiments are performed at two different constant forces: 5 pN (blue) and 15 pN (yellow). Fits to a double exponential function are shown in gray.

S5. Three-state kinetic model

We propose a model in which the binding reaction has an on-pathway intermediate state in which only one of the two intercalating moieties is bound to DNA (Figure 6**a**, main text). The model considers two different rates for binding: one rate corresponding to binding one chromophore when the ligand is unbound ($\alpha_{\rm on}$) that leads to the mono-intercalated intermediate state. This rate is expected to be concentration-dependent. Then there is a second binding rate ($\alpha'_{\rm on}$) that corresponds to intercalate the second moiety (once Thiocoraline is in the mono-intercalated intermediate state) in order to reach the fully bis-intercalated state. This kinetic rate is expected to be independent of ligand concentration. Finally, we consider that there is a third kinetic rate ($\alpha_{\rm off}$) that corresponds to the rate at which any of the intercalating moieties de-intercalates. For simplicity reasons, we considered a minimal model in which this unbinding rate is the same both to remove an intercalating moiety from the fully bound bis-intercalated state, or the mono-intercalated intermediate state.

The kinetics of the model can be described by means of the following master equations:

$$\frac{dP_{++}}{dt} = \alpha'_{\rm on}(P_{+-} + P_{-+}) - 2\alpha_{\rm off}P_{++} \quad \text{(S1a)}$$
$$\frac{dP_{+-}}{dt} = P_{--}\alpha_{\rm on} + P_{++}\alpha_{\rm off} - (\alpha_{\rm off} + \alpha'_{\rm on})P_{+-} \quad \text{(S1b)}$$
$$\frac{dP_{-+}}{dt} = P_{--}\alpha_{\rm on} + P_{++}\alpha_{\rm off} - (\alpha_{\rm off} + \alpha'_{\rm on})P_{-+} \quad \text{(S1c)}$$
$$\frac{dP_{--}}{dt} = \alpha_{\rm off}(P_{+-} + P_{-+}) - 2\alpha_{\rm on}P_{--} \quad \text{(S1d)}$$

where P_{++}, $P_{+-/-+}$ and P_{--} correspond respectively to the probabilities of a site to be: in the fully bound state (bis-intercalated), in the intermediate state (mono-intercalated), or non-occupied (naked DNA). The occupancy of the intermediate state can be grouped by defining $P_1 = P_{+-} + P_{-+}$:

$$\frac{dP_{++}}{dt} = \alpha'_{\rm on}P_1 - 2\alpha_{\rm off}P_{++} \quad \text{(S2a)}$$
$$\frac{dP_1}{dt} = 2P_{--}\alpha_{\rm on} + 2P_{++}\alpha_{\rm off} - (\alpha_{\rm off} + \alpha'_{\rm on})P_1 \quad \text{(S2b)}$$
$$\frac{dP_{--}}{dt} = \alpha_{\rm off}P_1 - 2\alpha_{\rm on}P_{--} \quad \text{(S2c)}$$

S5.1. Determining the microscopic rates $\alpha'_{\mathbf{on}}$ and $\alpha_{\mathbf{off}}$ from wash-off experiments

For wash-off experiments it can be assumed that α_{on}=0 (as no free-ligand is present in the buffer), and the set of equations can be analytically solved by finding the eigenvalues and eigenvectors of the reduced system:

$$\begin{pmatrix} P_{++} \\ P_1 \end{pmatrix} = \begin{pmatrix} -2\alpha_{\text{off}} & \alpha'_{\text{on}} \\ 2\alpha_{\text{off}} & -(\alpha_{\text{off}} + \alpha'_{\text{on}}) \end{pmatrix} \begin{pmatrix} P_{++} \\ P_1 \end{pmatrix} \tag{S3}$$

The eigenvalues of this system are determined as:

$$\lambda_{+-} = \frac{-(3\alpha_{\text{off}} + \alpha'_{\text{on}}) \pm \alpha_{\text{off}}\sqrt{1 + 6\alpha_{\text{off}}/\alpha'_{\text{on}} + (\alpha_{\text{off}}/\alpha'_{\text{on}})^2}}{2} \tag{S4}$$

The system therefore shows two characteristic timescales (a faster one, λ_- and a slower one λ_+) that can be directly related to the two off-rates observed in the wash-off experiments ($k_{\text{off,fast}}, k_{\text{off,slow}}$), finding that:

$$\alpha_{\text{off}} = \sqrt{\frac{k_{\text{off,fast}} k_{\text{off,slow}}}{2}} \tag{S5a}$$

$$\alpha'_{\text{on}} = k_{\text{off,fast}} + k_{\text{off,slow}} - 3\sqrt{\frac{k_{\text{off,fast}} k_{\text{off,slow}}}{2}} \tag{S5b}$$

From the experimental values $k_{\text{off,fast}}$ and $k_{\text{off,slow}}$ at different forces (Figure 5**b**, main text) we can determine the force-dependence of the intrinsic rates $\alpha'_{\text{on}}(F)$ and $\alpha_{\text{off}}(F)$ (Figure 6**b**, main text). Using these rates, the master equations can be solved for any set of initial conditions ($P_{++}(0)$, $P_1(0)$ and $P_{--}(0)$), finding solutions that will be of the form:

$$P_{++/1/--}(t) = A_{\text{fast}} e^{-k_{\text{off,fast}} t} + A_{\text{slow}} e^{-k_{\text{off,slow}} t} + A_\infty \tag{S6}$$

where A_{fast} and A_{slow} are the amplitudes of the fast and slow relaxation modes.

S5.2. Comparing the values of the amplitude as predicted by the model with the results from wash-off experiments.

In our experiments, we have access to the change in molecular extension as bis-intercalators bind or unbind. From the model depicted in Figure 6**a** (main text), the change in molecular extension $x(t)$ can be related to the probability of the bis-intercalated and intermediate states as:

$$x(t) \propto P_{++}(t) + f P_1(t) = A_{\text{off,fast}} e^{-k_{\text{off,fast}} t} + A_{\text{off,slow}} e^{-k_{\text{off,slow}} t} \tag{S7}$$

where we have assumed that the intermediate state contributes to a fractional extension, f, of that corresponding to a fully bound bis-intercalator (as we will later see $f \sim 0.4$ maximizes the agreement between model and experiments). Consequently, it is useful to define the normalized fractional elongation of the molecule $\chi(t)$, that goes from 0 (naked DNA) to 1 (maximal elongation due to full bis-intercalation):

$$\chi(t) = P_{++}(t) + 0.4P_1(t) \tag{S8}$$

For the wash-off experiments, the kinetics of the system (Eq. S2) can be analytically solved using the $\alpha'_{\text{on}}(F)$ and $\alpha_{\text{off}}(F)$ values shown in Figure 6**b** (main text). By assuming, that the molecule is initially in the fully bis-intercalated state ($P_{++}(0) = 1$, $P_1(0) = P_{--}(0) = 0$), we can determine the expected amplitude of the slow and fast decay rates ($A_{\text{off,fast}}$ and $A_{\text{off,slow}}$). The ratio between these amplitudes ($A_{\text{off,slow}}/(A_{\text{off,slow}}+A_{\text{off,fast}})$), can be compared to the experimental values obtained, finding compatible results (Figure 6**c** and Figure S7). The agreement between theory and experiment is maximized for an intermediate state that has an extension 0.4 times that of a fully bound bis-intercalator (Figure S7). This value is close to half that of a fully bound bis-intercalator, supporting the hypothesis that the intermediate state is mono-intercalated.

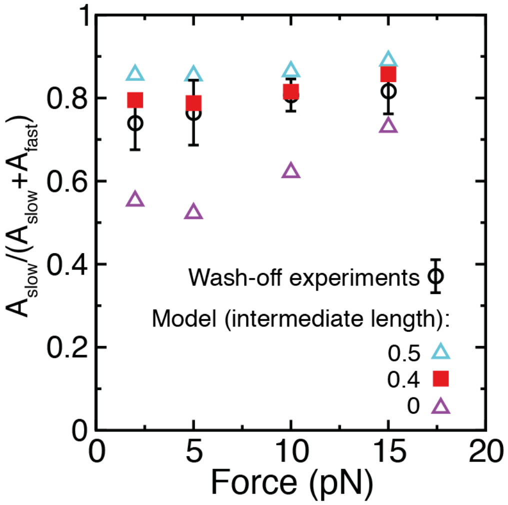

Figure S7. **Extension of the intermediate state.** The experimental measurement of the amplitude of the fast and slow kinetic rates observed in the wash-off experiments (black) is compared to the three-state kinetic model. The model best fits the experimental data if the length of the intermediate state is assumed to be 0.4 times that of a fully bis-intercalated ligand.

S5.3. Reproducibility of force-jump experiments and intercalative experiments starting from naked DNA ($\alpha_{\mathbf{on}} \neq 0$):

We performed two sets of experiments in which we monitored the binding kinetics: force-jump experiments started from an intercalated DNA equilibrated at low force, and constant-force intercalation starting with an initially naked DNA molecule. To model both types of binding experiments, we used the values of $\alpha'_{\text{on}}(F)$ and $\alpha_{\text{off}}(F)$ determined in the wash-off experiments (Figure 6**b**, main text).

To convert the molecular extension measured in the experiments to the fractional elongation $\chi(t)$, we assumed a maximum binding density ($n = 4$) of bis-intercalated ligands, with each bis-intercalated ligand elongating DNA by $\Delta l_{0,\text{Thio}} = 0.68$ nm:

$$\chi(t) = \frac{x_{\text{Thio}}(t,F) - x_{\text{DNA}}(F)}{(N_{\text{bp}}\Delta l_{0,\text{Thio}}/n)\phi_{F,\text{WLC}}} \simeq \frac{2(x_{\text{Thio}}(t,F) - x_{\text{DNA}}(F))}{x_{\text{DNA}}(F)} \tag{S9}$$

where N_{bp} is the number of base pairs of the DNA molecule, n is the binding density (=4) and $\phi_{F,\text{WLC}}$ a WLC correction to the molecular extension due to the force applied to the molecule. For this correction we used the fact that the elastic properties of intercalated DNA are similar to those of naked DNA, and therefore $N_{\text{bp}}\Delta l_{0,\text{Thio}}\phi_{F,\text{WLC}} \sim 2x_{\text{DNA}}(F)$ obtaining the simplified expression.

For the force-jump experiments, as far as $\alpha_{\text{on}}(F) \geq \alpha'_{\text{on}}(F)$, the experiments are well described. In these experiments, we assumed that the molecule starts in equilibrated conditions at 2 pN ($dP_{++}/dt = dP_1/dt = dP_{--}/dt = 0$) with rates $\alpha_{\text{off}}(F = 2\text{ pN})$, $\alpha'_{\text{on}}(F = 2\text{ pN})$ and $\alpha_{\text{on}}(F = 2\text{ pN})$. The experiment starts with a force-jump in which these kinetics rates are changed, and therefore the master equations are solved by considering the previous initial conditions and the new kinetic rates at each force (15-55pN). The values of $\alpha_{\text{off}}(F)$ and $\alpha'_{\text{on}}(F)$ are determined using an extrapolation of the wash-off experiments (blue and red regions in Figure 6**b**, main text). In this situation α'_{on}(F), is the rate-limiting step of the reaction, explaining the fact that force-jump experiments show little variation when the concentration is increased from 100 nM to 1 μM.

Finally, we assessed the binding kinetics for experiments in which the initial conditions correspond to naked DNA ($P_{--} = 1$), with the determined kinetic rates. In this case, the binding kinetics are well described considering an α_{on} that increases with force with a similar slope as for α'_{on} (Figure 6**b**, main text). These values are found to be compatible with the

results obtained in the force-jump experiments.

S6. Single-molecule footprinting of preferred binding sites

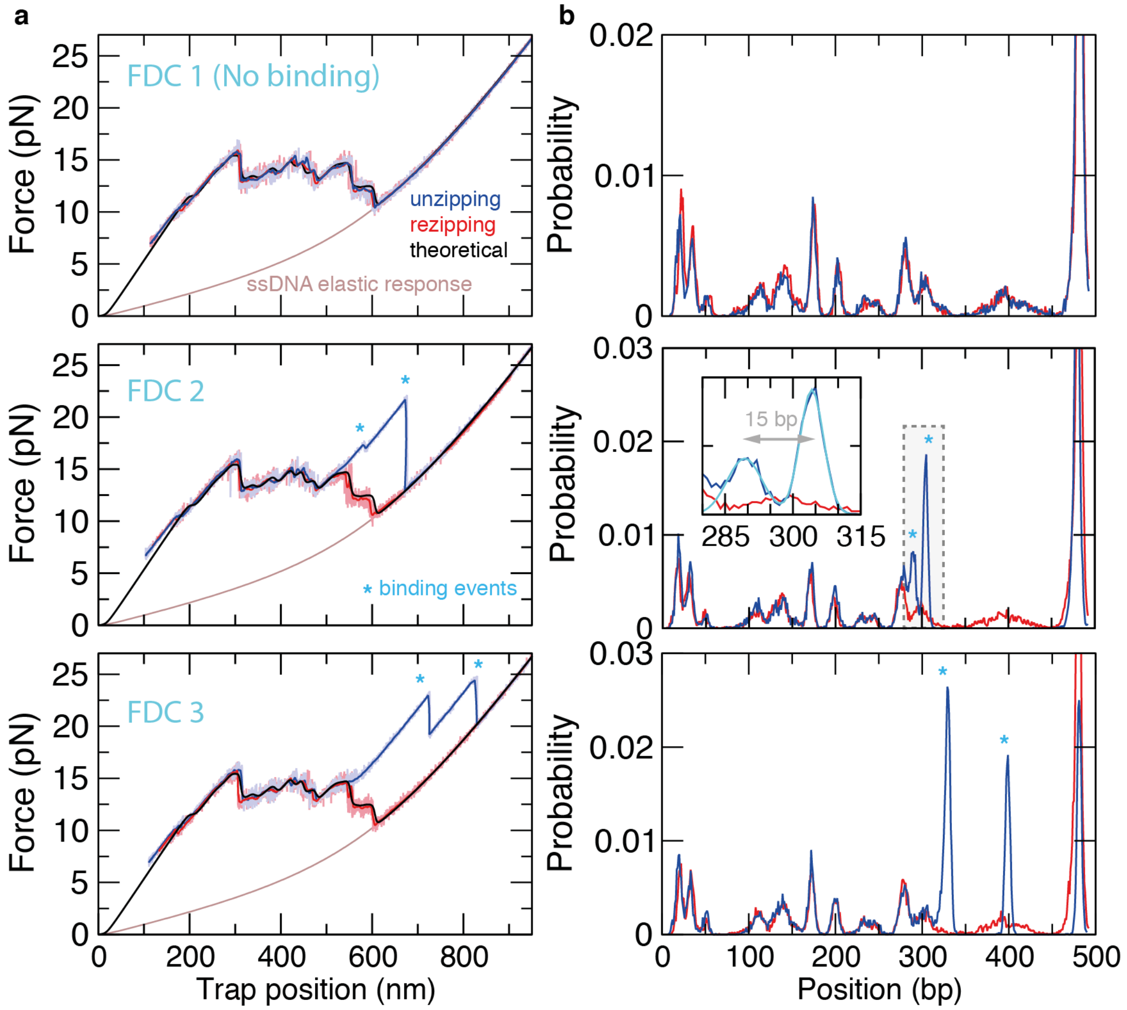

Figure S8. **Single-molecule footprinting of Thiocoraline binding.** (**a**) Set of three FDCs showing none (top) and 2 (middle and bottom) binding events. Pulling speed is 70 nm/s. At this speed the unzipping (blue) and rezipping (red) curves are fully reversible in the absence of binding events (top). FDC's are aligned to a theoretical curve (black), and a FJC curve of a fully unfolded molecule (480 bp) is plotted as a reference (brown). If an individual Thiocoraline molecule binds to the hairpin, its binding site can be identified from the position of the observed force peak (indicated with an *, see methods). Data is obtained at 1 kHz (light blue and red), and a 10 Hz average shown in dark colours. (**b**) Histogram of the number of open base pairs (n) of the FDCs shown in panel (a). As expected, the histograms of the unzipping (blue) and rezipping (red) curve fully overlap for pulling cycles without binding events (top). Binding events are easily identified in the unzipping histogram as peaks that do not have a counterpart in the rezipping curve (middle and bottom, *). These peaks are well described by a Gaussian distribution (cyan, middle inset), and the binding positions determined from its mean. Note for instance, that two binding events closely spaced in the same FDC (middle, inset) can be easily resolved with this technique.

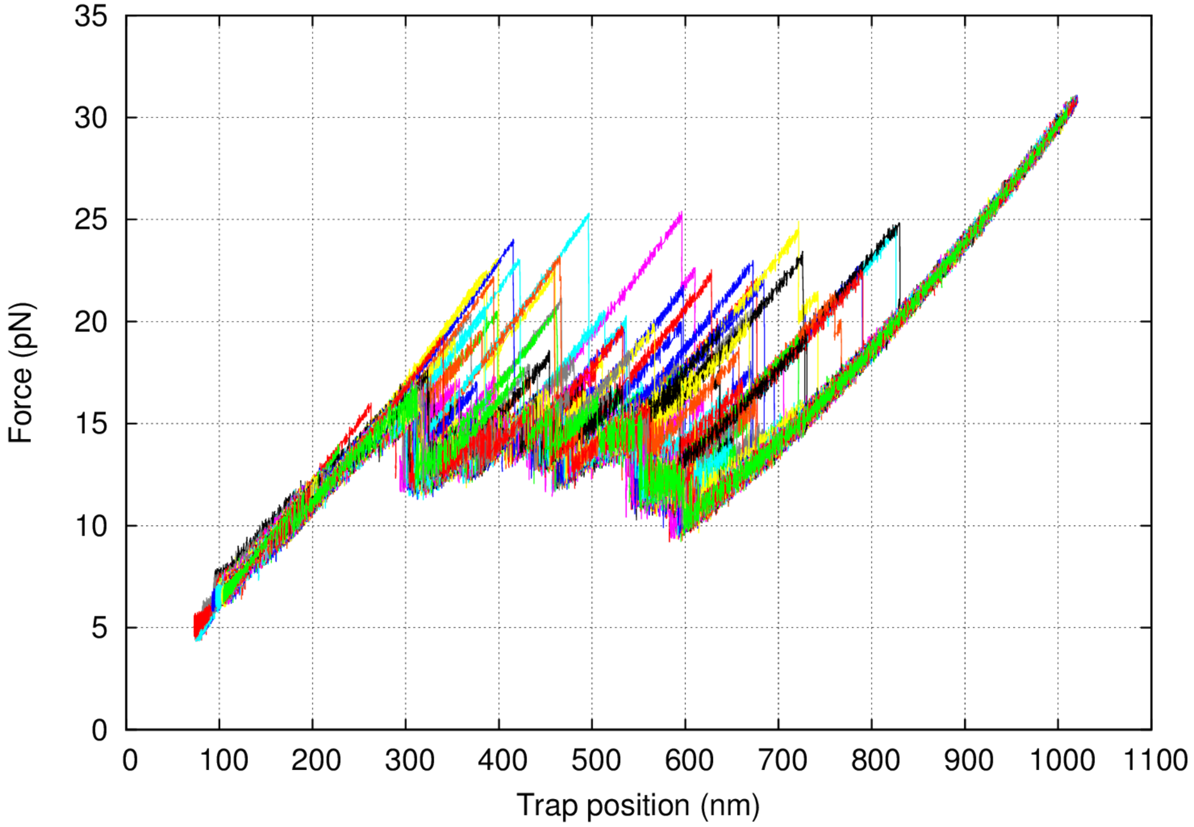

Figure S9. **Consecutive unzipping curves in the presence of Thiocoraline.** In this figure several DNA unzipping curves are shown superimposed using a different color for each curve. Each unzipping curve contains a few binding events (typically between 0 and 2). By overlapping a large number of unzipping curves it can be seen that the ligand binds to many positions along the DNA sequence. Statistics on the preferred binding sites of the ligand are obtained by collecting a large number of unzipping curves ($N > 100$) where the different binding sites become populated.

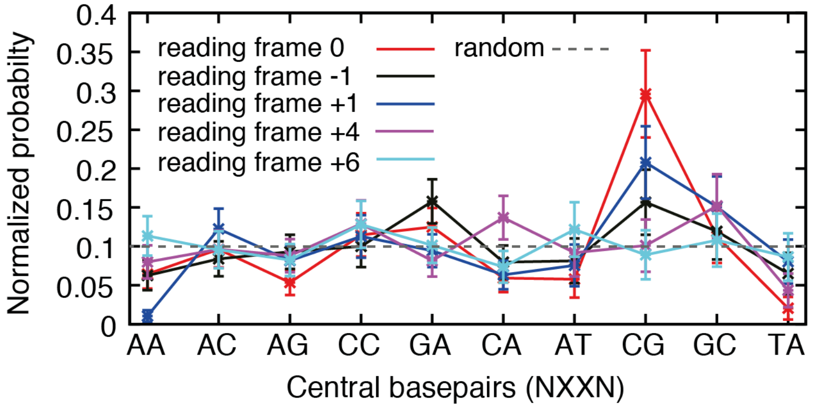

Figure S10. **Sequence-selectivity of Thiocoraline clamping positions.** Normalized probability of observing a binding event in which Thiocoraline clamps the central dinucleotide step XX (of a tetra nucleotide motif NXXN) if different reading frames are considered. Binding at the tetranucleotide position were blockage is observed (reading frame 0, red) is compared to alternative distances from the peak position (black, blue, purple, cyan). Sequence-selectivity is only observed for the reading frame 0, whereas other reading frames show probabilities compatible with a random distribution (dashed gray). Only for the reading frames ±1 (black and blue) a lower peak at CG is also observed, indicative of the error associated with the method.

[S1] Vladescu, I. D., McCauley, M. J., Rouzina, I., and Williams, M. C. (2005) *Phys. Rev. Lett.* **95(15)**, 158102.

[S2] Murade, C., Subramaniam, V., Otto, C., and Bennink, M. L. (2009) *Biophys. J.* **97(3)**, 835–843.

[S3] Fu, H., Chen, H., Marko, J. F., and Yan, J. (2010) *Nucleic Acids Res.* **38(16)**, 5594–5600.

[S4] King, G. A., Gross, P., Bockelmann, U., Modesti, M., Wuite, G. J., and Peterman, E. J. (2013) *Proc. Natl. Acad. Sci. USA* **110(10)**, 3859–3864.
